# Tumor-Resident Dendritic Cells and Macrophages Modulate the Accumulation of TCR-Engineered T Cells in Melanoma

**DOI:** 10.1016/j.ymthe.2018.03.011

**Published:** 2018-03-16

**Authors:** Alastair Hotblack, Angelika Holler, Alice Piapi, Sophie Ward, Hans J. Stauss, Clare L. Bennett

**Affiliations:** 1 Institute for Immunity and Transplantation, Division of Infection and Immunity, University College London, London NW3 2PF, UK; 2 Cancer Institute, Division of Cancer Studies, University College London, London WC1E 6DD, UK

**Keywords:** dendritic cells, myeloid cells, engineered T cells, adoptive cellular therapy, tumor

## Abstract

Ongoing clinical trials explore T cell receptor (TCR) gene therapy as a treatment option for cancer, but responses in solid tumors are hampered by the immunosuppressive microenvironment. The production of TCR gene-engineered T cells requires full T cell activation *in vitro*, and it is currently unknown whether *in vivo* interactions with conventional dendritic cells (cDCs) regulate the accumulation and function of engineered T cells in tumors. Using the B16 melanoma model and the inducible depletion of CD11c^+^ cells in CD11c.diphtheria toxin receptor (DTR) mice, we analyzed the interaction between tumor-resident cDCs and engineered T cells expressing the melanoma-specific TRP-2 TCR. We found that depletion of CD11c^+^ cells triggered the recruitment of cross-presenting cDC1 into the tumor and enhanced the accumulation of TCR-engineered T cells. We show that the recruited tumor cDCs present melanoma tumor antigen, leading to enhanced activation of TCR-engineered T cells. In addition, detailed analysis of the tumor myeloid compartment revealed that the depletion of a population of DT-sensitive macrophages can contribute to the accumulation of tumor-infiltrating T cells. Together, these data suggest that the relative frequency of tumor-resident cDCs and macrophages may impact the therapeutic efficacy of TCR gene therapy in solid tumors.

## Teaser

The extent to which tumor-specific engineered T cells are regulated by endogenous immune cells is unclear. Here, Hotblack et al. exploit depletion of CD11c-expressing myeloid cells to demonstrate that dynamic interactions within the tumor microenvironment control the expansion of engineered T cells.

## Introduction

The success of cancer immunotherapy in recent years has changed the way we treat cancer. Therapies that target endogenous adaptive immune responses against tumors in patients, such as antibody blockade of inhibitory pathways that prevent T cell function, have led to tumor remission in patients with previously untreatable cancers. However, the non-specific nature of this approach may lead to serious side effects, due to the release of auto-reactive T cells. One alternative to enhance anti-tumor immunity and reduce auto-reactivity is redirecting the specificity of T cells using T cell receptors (TCRs) or chimeric antigen receptors (CARs). The transfer of TCR-modified T cells specific for melanoma-associated antigens has been shown to lead to clinical responses, including durable tumor regression in a subset of patients.^1^, ^2^, ^3^ However, clinical responses to gene-modified T cells for solid tumor cancers have not shown the success of clinical trials for hematologic malignancies. Improving treatments requires a more detailed understanding of the cellular interactions that modify T cell function in solid tumors.

Tumors are populated by different populations of myeloid cells that express the integrin CD11c. Of these, conventional dendritic cells (cDCs) are specialized antigen-presenting cells that control T cell immunity. Lineage tracing experiments in mice have mapped two developmentally and functionally distinct populations, cDC1 and cDC2, that reside in peripheral tissues where they are defined by expression of CD103 and CD11b, respectively.^1^ These lineages and their functions are conserved in humans.^2^, ^3^, ^4^ Of these, cDC1 are highly efficient at cross-presenting antigens to cytotoxic T cells and are the major stimulatory cDC population within tumors, both for the generation of anti-tumor immunity in draining lymph nodes (LNs) and upon direct interaction with effector T cells within the tumor microenvironment.^5^, ^6^, ^7^, ^8^ Furthermore, cDC1 are critical for therapeutic responses to checkpoint blockade,^9^, ^10^, ^11^ but their possible role in modifying the function of engineered T cells has not been as extensively studied.

Engineering T cells to express TCRs specific to tumor antigens commonly uses retro- or lentiviral gene transfer vectors. Transduction with these vectors depends on T cell activation and proliferation, which is required for access of genes encoded in the vector into the nucleus for integration into the host genome. As such, T cells are routinely activated *in vitro* using non-specific mitogens, or stimulation of CD3 and CD28 as part of the transduction protocol, and transferred as effector-like T cells into hosts. An advantage of this strategy is that transfer of activated T cells circumvents priming by cDCs, which may be dysfunctional in the cancer patient.^12^ But direct interactions between tissue DCs and effector or memory T cells outside secondary lymphoid organs are also required for T cell function and survival,^13^ and, within the tumor, cDCs directly interact with effector T cells.^8^, ^14^ In addition to cDCs, tumors contain populations of monocytes and macrophages that express varying levels of CD11c, which are commonly associated with the development of an immunosuppressive tumor environment through secretion of cytokines such as interleukin 10 (IL-10) or transforming growth factor β (TGF-β).^15^ However, the extent to which the number and function of transduced T cells is affected by CD11c^+^ cells once they are recruited to the tumor is not known.

In this study, we have exploited an inducible model of CD11c^+^ cell depletion to investigate the impact of CD11c^+^ cells, including cDCs, on the fate of T cells engineered to express an H2-K^b^-restricted TCR against the melanoma-associated antigen tyrosinase-related protein 2 (TRP-2).^16^ We demonstrate that dynamic interactions with different myeloid cells control accumulation of transferred T cells within the changing tumor environment. Depletion of CD11c^+^ cells triggered the recruitment of cross-presenting cDC1 into the tumor and a loss of CD11c^+^ macrophages, resulting in the accumulation of TRP-2 TCR-engineered T cells. Together, these data indicate that the balance between tumor-resident cDCs and macrophages impacts the accumulation of TRP-2 TCR-engineered T cells in B16 tumors.

## Results

### Characterizing Depletion of CD11c^+^ Cells from B16 Tumors in CD11c.DTR Mice

**Figure 1:**
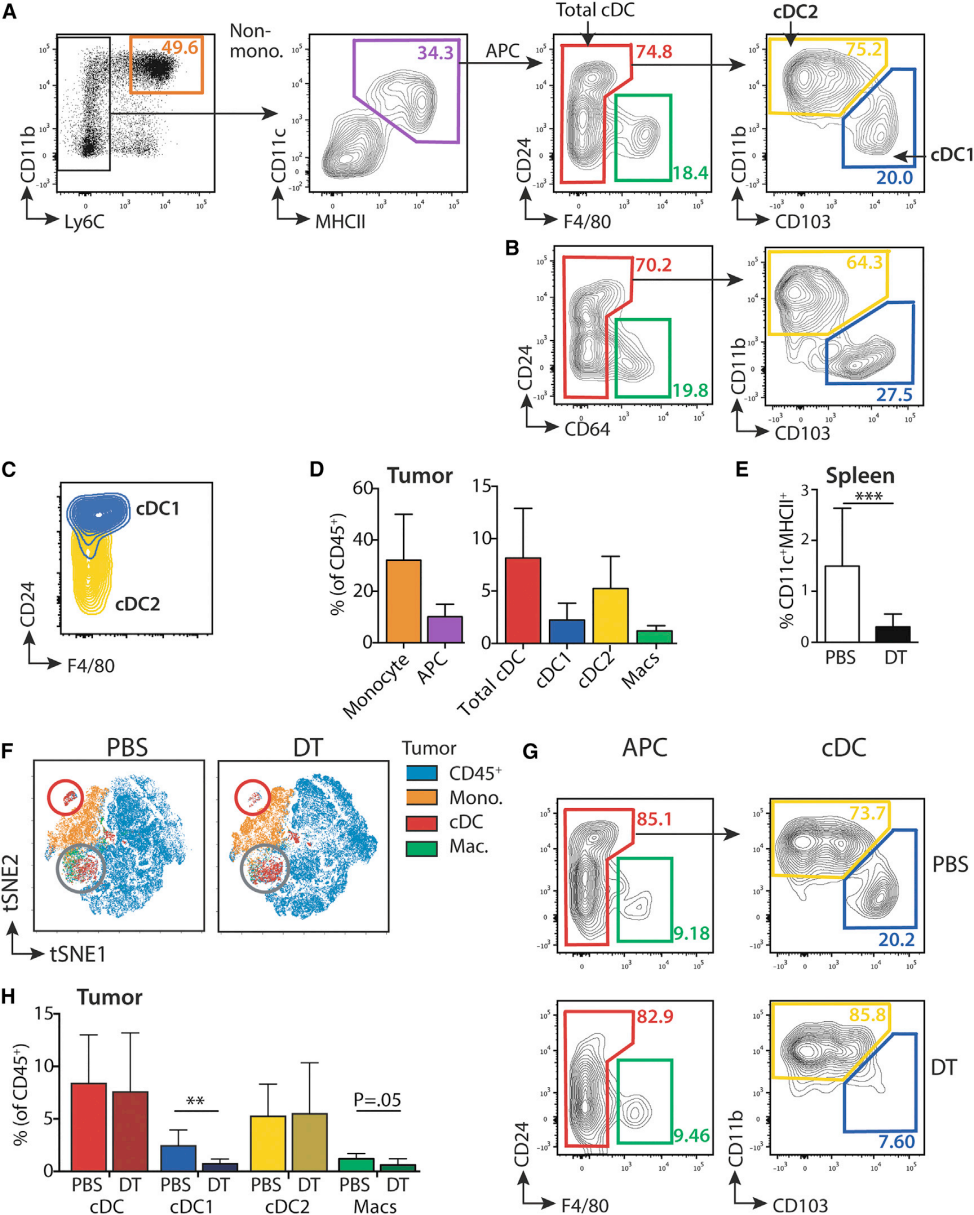
Characterizing Depletion of CD11c^+^ Cells from B16 Tumors (A) Mice were injected subcutaneously (s.c.) with 5 × 10^5^ B16.F10 cells, and tumors were harvested 17 days later. Representative flow cytometric plots show the gating of myeloid and cDC populations within tumor-infiltrating leukocytes. Plots are pre-gated on live, single CD45^+^ cells and shown exclusion of Ly6C^+^ monocytes (non-mono) to produce an APC (antigen presenting cell) population that contains F4/80^+^ macrophages (macs) and cDC populations. (B) Representative plots from an independent experiment demonstrating that use of a flow cytometry panel in which macrophages are identified with CD64 results in similar population frequencies. (C) Representative contour plots showing expression of CD24 and F4/80 by pre-gated cDC1 (blue) and cDC2 (yellow). (D) Summary bar graph showing mean frequency of myeloid cell populations as a proportion of total CD45^+^ cells in the tumor (±SD); n = 8, from two independent experiments. Mono., monocyte; Macs, F4/80^+^ macrophages. (E) CD11c.DTR mice were injected intraperitoneally (i.p.) with 100 ng DT. Bar chart shows the frequency of splenic CD11c^+^/MHCII^+^ cDCs (±SD) 48 hr later. p = 0.0002; n = 6, from >3 independent experiments. (F) Nonlinear dimensionality reduction analysis of CD45^+^ cells in d17 tumors 48 hr after injection of PBS or DT. t-SNE maps are based on the parameters CD11c, MHCII, Ly6C, CD103, CD24, CD11b, and F4/80, and colors indicate overlays of different populations as shown in (A), on the tSNE coordinates. Data are pooled from 4 samples from 1 representative experiment. The red circle marks the cDC1 cluster, while gray circles highlight cDC2 and macrophages. (G) Representative contour plots showing tumor cDCs 48 hr post-injection of DT and (H). Summary bar graph of the data (±SD). p = 0.008; n = 8, from three independent experiments.

As an initial approach to dissect the role of tumor-resident CD11c^+^ populations in the activation of TCR gene-modified T cells, we analyzed the inducible depletion of cDCs, and other CD11c^+^ cells, 48 hr after injection of diphtheria toxin (DT) into CD11c.diphtheria toxin receptor (DTR) mice bearing subcutaneous B16 tumors. Tumors were digested 17 days post-injection, at which point they had reached approximately 75 mm^2^. To identify tumor cDCs by flow cytometry, we excluded Ly6C^+^ monocytes, and analyzed CD11c^+^MHCII^+^ cells, which were either F4/80^neg^ or CD64^neg^ (Figures 1A and 1B). Expression of CD24 distinguishes conventional cells from monocyte-derived cells.^17^ Within the CD24^low to high^ cDC population, cDC1 were defined by expression of CD103^+^ and high levels of CD24, while CD11b^+^ cDC2 expressed low to intermediate levels of CD24 (Figure 1C). Therefore, to include both populations, we used a broad CD24 gate in this study. Figures 1A–1D show that cDCs in B16 tumors were largely comprised of cDC2, with cDC1 representing a smaller subset, in agreement with published data.^18^ Injection of DT into CD11c.DTR recipients led to the depletion of all CD11c^+^ cDCs from the spleen within 48 hr (Figure 1E). To objectively assess the impact of DT on tumor immune cells, we exploited an unsupervised analysis using multidimensional reduction analysis of flow cytometry data. Figure 1F shows viSNE maps, which allow visualization of the data derived from the t-distributed stochastic neighbor embedding (t-SNE) algorithm.^19^ Here, pre-defined myeloid cell populations were overlaid onto the t-SNE plot for total CD45^+^ cells. Using this analysis, cDC1 could be distinguished as a distinct cluster of cells, which was lost from tumors in DT-treated mice, (Figure 1F, see red circled population). By comparison, cDC2 and macrophages were displayed as merged clusters and appeared less affected by a single injection of DT (Figure 1F, gray circles). Analysis of the relative frequencies of these populations within CD45^+^ cells using flow cytometric plots demonstrated that cDC1 were highly sensitive to a single injection of DT, while cDC2 were not depleted. We observed a trend toward depletion of F4/80^+^ macrophages after DT injection (Figures 1G and 1H). Having characterized baseline responses to DT, we subsequently investigated whether these changes in the endogenous tumor myeloid compartment affected the number and function of adoptively transferred TCR-engineered T cells.

### Depletion of CD11c^+^ Cells from CD11c.DTR Chimeras Leads to Enhanced Accumulation of Activated TCR-Engineered T Cells

**Figure 2:**
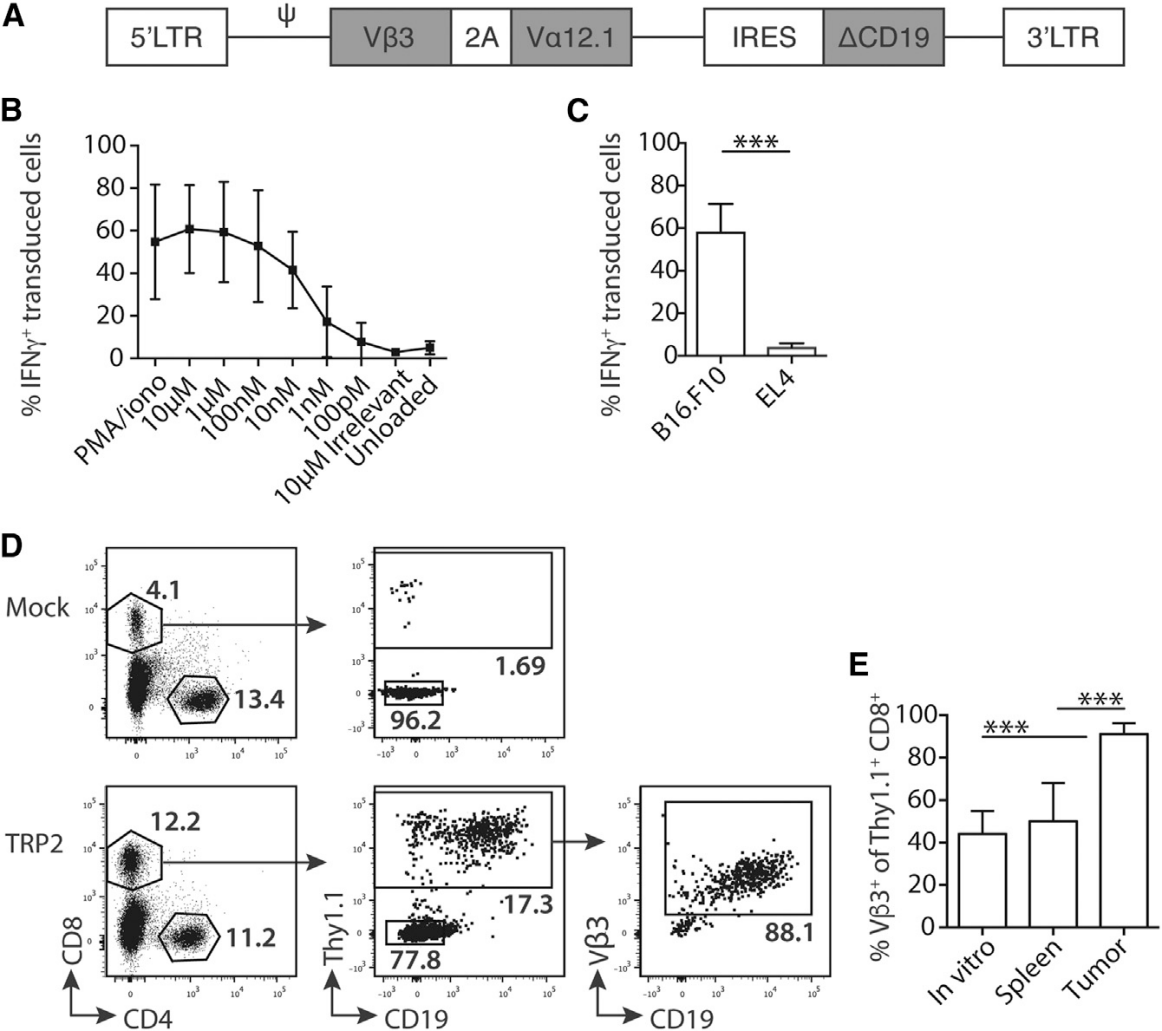
T Cells Engineered to Express TRP-2 TCRs Accumulate in B16 Tumors (A) A schematic showing the retroviral vector containing the TRP-2-TCR construct composed of Vα12.1 and Vβ3 TCR chains separated by a 2A sequence. CD19 with a truncated endodomain was used as a marker of transduction. LTR, long-terminal repeat; ψ, retroviral packaging element; IRES, internal ribosome entry site. (B) CD8^+^ TRP-2-TCR-transduced (CD19^+^) splenocytes were co-cultured overnight with TRP-2_181–188_ peptide, irrelevant peptide, or unloaded RMA/S cells. The line graph shows the mean frequency (±SD) of intracellular IFN-γ^+^-transduced T cells. (C) TRP-2-TCR-transduced T cells were co-cultured overnight with irradiated B16.F10 (tumor antigen positive) or EL4 (antigen negative) tumors. The bar chart shows a summary of the mean frequency (±SD) of IFN-γ^+^-transduced T cells, p = 0002. Data for (B) and (C) are pooled from 3–4 independent experiments, n = 4. (D) Representative dot plots show the gating strategy for identification of transferred (CD8^+^ Thy1.1^+^) lymphocytes from the tumor. Cells are pre-gated on live, single CD45^+^ cells. TRP-2-TCR-transduced cells were further identified by expression of CD19 or Vβ3. Mock transduced cells were cultured without the addition of retrovirus. (E) Summary bar graph showing the mean frequency (±SD) of transduced cells (based on Vβ3 expression) from *in vitro* cells before injection or from the CD8^+^ Thy1.1^+^ cells isolated from the spleen or tumor 13 days post-transduction, n = 4 (*in vitro*) or n = 11/13 (spleen/tumor, respectively). *In vitro* cells versus tumor and spleen versus tumor. p < 0.0001. Data are from three independent experiments. ^∗∗∗^p < 0.001.

To test a requirement for CD11c^+^ cells, in particular cDC1, on adoptively transferred tumor-specific T cells, we established a model of adoptive cell therapy (ACT) in which CD8^+^ T cells were engineered to express the anti-melanoma TCR, which recognizes TRP-2_181–188_ presented by H2-K^b^.^20^ Murine CD8 T cells were activated by ConA and IL-7 and transduced with a retroviral vector encoding the TRP-2-specific TCR (Figure 2A). Transduced T cells were peptide specific and recognized B16 tumor cells expressing TRP-2 endogenously, but not H2-Kb^+^ EL4 tumors that did not express TRP-2 (Figures 2B and 2C). 4 × 10^6^ CD8^+^ Thy1.1^+^ T cells, containing on average 1.8 × 10^6^ ± 0.2 × 10^6^ (SEM) TRP-2-specific T cells, were transferred, without preconditioning, to mice bearing established B16.F10 tumors. This resulted in the antigen-specific accumulation of transferred T cells *in vivo* (Figure 2D). Transferred T cells were identified by expression of the congenic marker Thy1.1, the reporter gene CD19, and the TRP-2-specific Vβ3 chain. After transfer, TRP-2-specific T cells accumulated within B16 tumors. For example, before injection, approximately 40% of transferred T cells expressed the TRP-2 TCR, and, 12 days after adoptive transfer, this frequency was largely unchanged in the spleen of recipient mice. In contrast, the analysis of tumor-infiltrating lymphocytes showed that >90% of adoptively transferred Thy1.1^+^ T cells in the tumor expressed the TRP-2 TCR, indicating selective enrichment of TCR-expressing T cells in the tumor microenvironment (Figure 2E).

**Figure 3:**
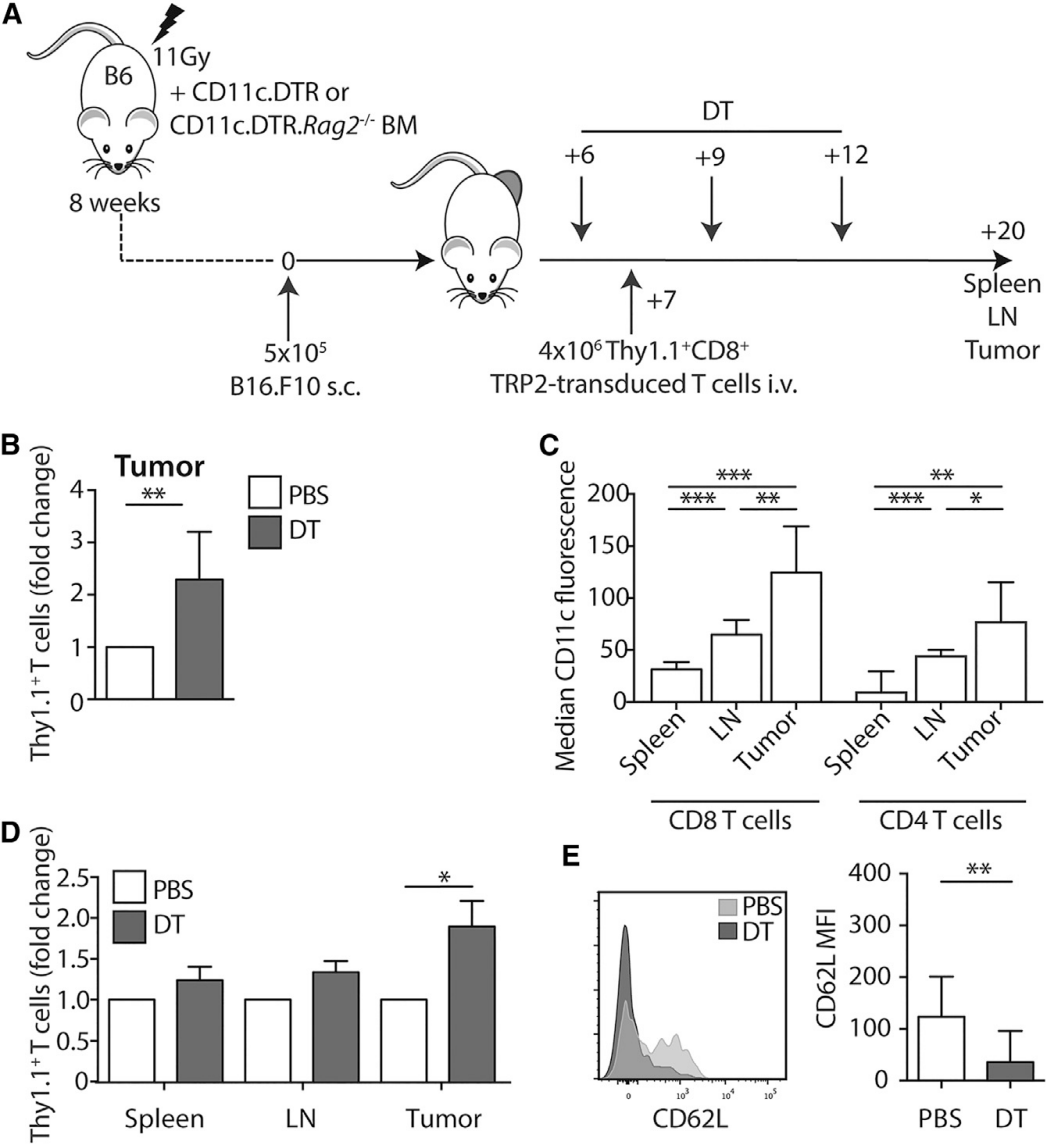
Depletion of CD11c^+^ Cells Leads to Enhanced Expansion of Tumor-Specific T Cells (A) Schematic showing the experiment. CD11c.DTR or CD11c.DTRx*Rag2*^−*/*−^ into syngeneic C57BL/6 chimeras were established and injected s.c. with B16.F10 cells. Mice were depleted of CD11c^+^ cells by injection of 100 ng DT on days 6, 9, and 12. 1 day after the first dose of DT, mice also received 4 × 10^6^ Thy1.1^+^ CD8^+^ T cells that had been transduced with the TRP-2 TCR. Mice were sacrificed on day 20, and transferred cells were analyzed in spleens, LNs, and tumors. (B) Summary bar graph showing the fold change in frequency of tumor-infiltrating Thy1.1^+^ T cells from total CD8 in DT-treated CD11c.DTR chimeras (gray) compared to the mean of the PBS control group (white) (±SD). p = 0.0156; n = 8 from two independent experiments. (C) Bar graph showing median CD11c fluorescent intensity (±SD) on CD4^+^ or CD8^+^ lymphocytes. CD8 spleen versus LN and spleen versus tumor p = 0.0001; LN versus tumor p = 0.005. CD4 spleen versus LN p = 0.001; spleen versus tumor p = 0.002; LN versus tumor p = 0.05; n = 7 from two independent experiments. (D) Summary bar graph showing fold change in frequency (±SD) of Thy1.1^+^ T cells from the total CD8 in CD11c.DTR.*Rag2*^−*/*−^ chimeras after treatment with DT (gray) or PBS (white). Tumor p = 0.0018; n = 15-19, from four independent experiments. (E) Left: representative histogram overlay showing expression of CD62L by tumor-infiltrating Thy1.1^+^ T cells in DT- or PBS-treated mice. Right: summary bar graph showing median CD62L fluorescence intensity (±SD) after treatment with PBS or DT. p = 0.0027; n = 12–15 from three independent experiments. ^∗^p < 0.05; ^∗∗^p < 0.01; ^∗∗∗^p < 0.001.

The activity of the CD11c promoter in CD11c.DTR transgenic mice leads to aberrant expression of the DTR in the brain and gut, leading to death of these mice after repeated injections of DT.^21^, ^22^ To bypass this effect, syngeneic chimeras are routinely generated in which the DTR transgene is restricted to the hematopoietic system. Therefore, to test the impact of the loss of CD11c^+^ cells on TRP-2-expressing T cells, we transferred TRP-2 TCR-engineered T cells into tumor-bearing established CD11c.DTR→B6 chimeras and depleted CD11c^+^ cells by injection of DT from 1 day before until 5 days after T cell transfer (Figure 3A). Mice were sacrificed 8 days after the last injection of DT. Figure 3B shows that injection of DT led to an increase in the relative frequency of adoptively transferred T cells in tumors. However, we observed that endogenous T cells in B16 tumors expressed high levels of CD11c, which might result in their depletion after DT treatment (Figure 3C). Local depletion of endogenous CD11c^+^ T cells could trigger a lymphopenia-induced expansion of the adoptively transferred DT-insensitive T cells. Therefore, in order to control for this possibility, we repeated our experiments by generating CD11c.DTR.*Rag2*^−/−^ mice and using bone marrow (BM) from these mice to generate syngeneic chimeras. Figure 3D demonstrates that DT-mediated depletion of CD11c^+^ cells from mice lacking DT-sensitive endogenous T cells also led to a relative increase in the frequency of transferred T cells in the tumor, but not spleen and LN. These data indicated that the accumulation of transferred T cells in the tumor microenvironment after DT treatment was not driven by the local depletion of endogenous T cells.

We further explored whether DT treatment affected the phenotype of the adoptively transferred T cells in the tumor. Figure 3E shows that the transferred T cells in the tumors of DT-treated recipients expressed lower levels of CD62L compared to PBS-treated controls, suggesting that depletion of CD11c^+^ cells was associated with a more differentiated CD62L^neg^ phenotype of TCR-engineered T cells.

Therefore, together, these data demonstrate that loss of myeloid CD11c^+^ cells leads to an enhanced accumulation of differentiated tumor-specific T cells within the tumor bed by mechanisms that are independent of endogenous lymphocytes.

### Depletion of cDCs Does Not Result in Accumulation of Monocytes within Tumors

**Figure 4:**
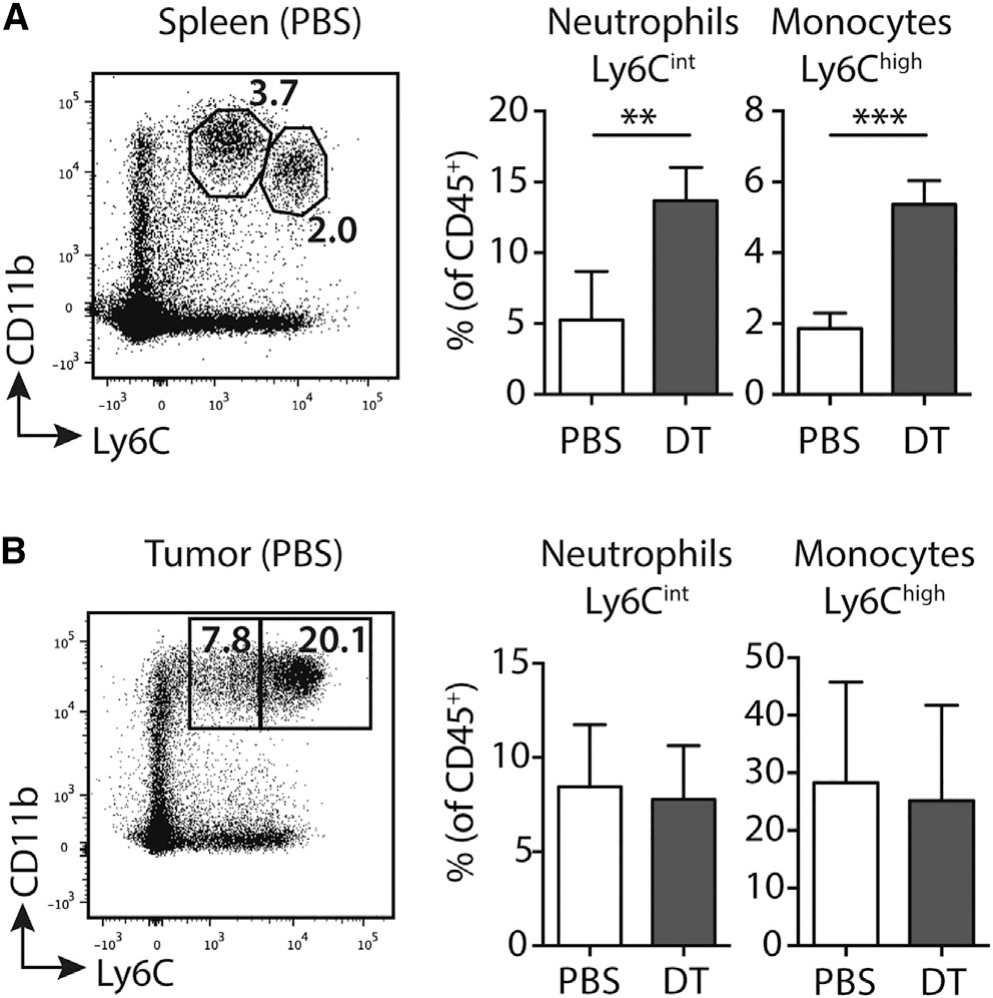
Depletion of CD11c^+^ Cells Does Not Lead to Accumulation of Tumor Monocytes Left: representative dot plots showing gating of CD11b^+^Ly6C^int^ neutrophils and CD11b^+^Ly6C^high^ monocytes in the spleen (A) and B16 tumor (B) of control mice. Plots are pre-gated on live CD45^+^ cells. Right: bar graph showing mean frequencies (±SD) of cells in the spleen (A) and tumor (B) site on day 14, 48 hr after the last injection of DT. Splenic neutrophils p = 0.007, monocytes p = 0.0001; n = 4 (spleen) or 8 (tumor) from three independent experiments. ^∗∗^p < 0.01; ^∗∗∗^p < 0.001.

Depletion of cDCs results in monocytosis and neutrophilia due to increased levels of serum granulocyte colony-stimulating factor (G-CSF).^23^, ^24^, ^25^ Therefore, to test whether increased numbers of monocytes (or monocyte-derived cells) could impact the accumulation of TRP-2 T cells, we analyzed the frequency of monocytes in the tumors of DT-injected mice. Figures 4A and 4B show that, despite a clear increase in the frequency of monocytes and neutrophils in the spleens of DT-injected CD11c.DTR chimeras, these cells did not accumulate in B16 tumors. These data are consistent with our previous finding that circulating monocytes did not migrate to the skin after depletion of cDCs.^24^ Therefore, these experiments demonstrate that the monocytosis and neutrophilia induced post-depletion of CD11c^+^ cells does not impact the local tumor environment, and, therefore, that expansion of T cells cannot be explained by an increase in tumor monocytes.

### Depletion of CD11c^+^ Cells Re-sets the Tumor cDC Niche

**Figure 5:**
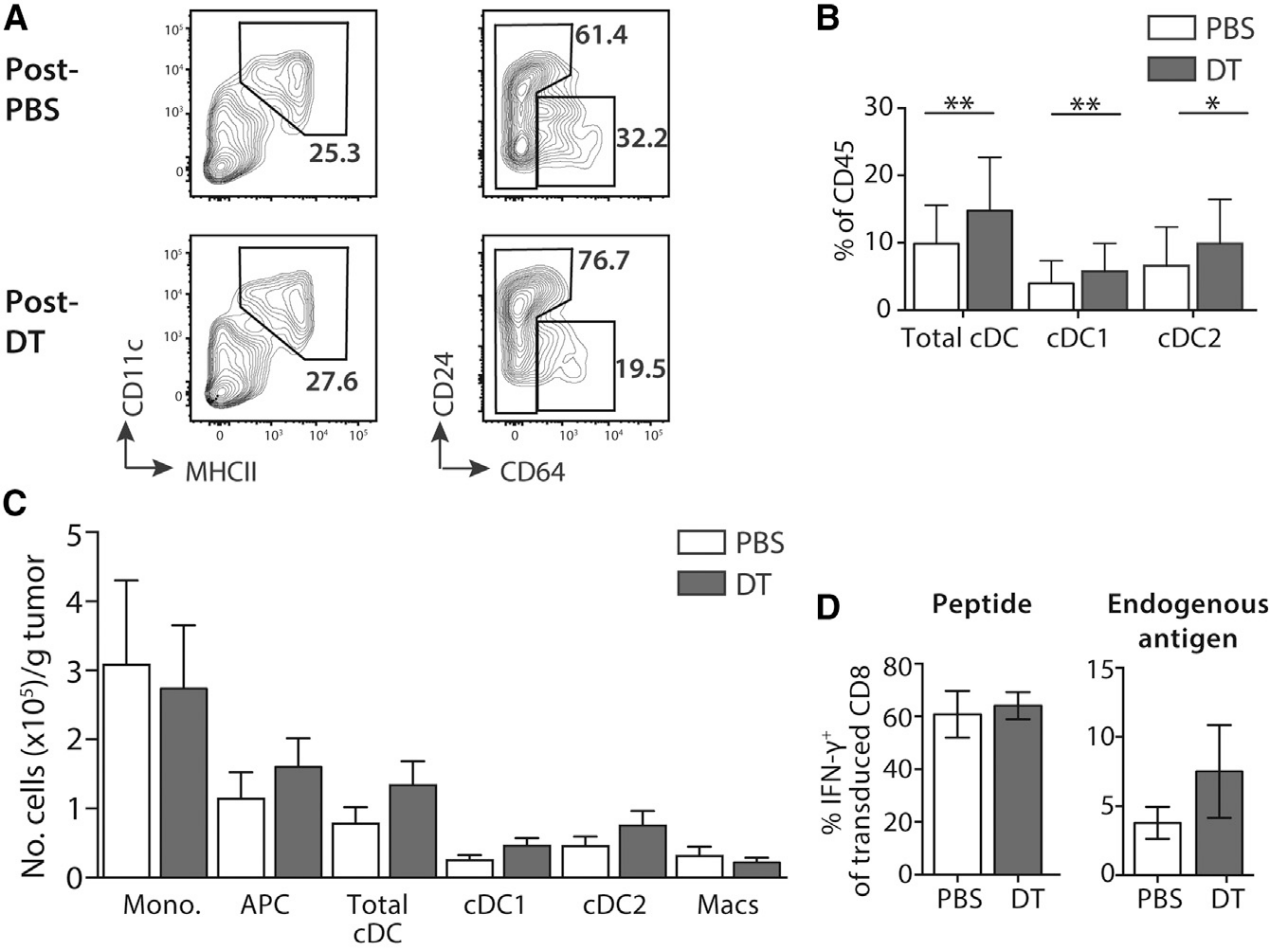
Depletion of CD11c^+^ Cells Results in Enhanced Accumulation of Tumor cDCs (A) Representative contour plots showing myeloid cell populations in B16 tumors 1 week after the last injection of DT (see also schematic in Figure 3). Plots were pre-gated on live, single CD45^+^Ly6c^neg^ cells. (B) Bar graph shows summary mean frequency (±SD) of cDCs, cDC1, or cDC2 of total CD45^+^ cells on day 19 in PBS- (white) or DT-treated (gray) recipients. PBS versus DT cDCs p = 0.009, cDC1 p = 0.005, cDC2 p = 0.04. (C) Bar graphs showing the mean number of cells (±SD) 1 week post-PBS or -DT. n = 16–18 from four independent experiments for frequency and numbers. (D) Bar charts showing the mean frequency (±SD) of intracellular IFN-γ^+^ cells from CD8^+^ TRP-2-TCR-transduced (CD19^+^) splenocytes. Cells were cocultured with peptide-pulsed or non-peptide-pulsed tumor-derived CD11c^+^ cells sorted on day 19. n = 3 per group, one representative experiment shown of two independent experiments. ^∗^p < 0.05; ^∗∗^p < 0.001.

In the experiments described above, mice were sacrificed 8 days after the last injection of DT (Figure 3A), during which time the DC niche would be expected to be repopulated by BM-derived cells.^22^ Therefore, we questioned whether emerging cDCs, which have not yet been subverted by the tumor microenvironment, might mediate expansion of tumor-specific T cells. To address this hypothesis, we first characterized the tumor myeloid compartment 1 week after the last injection of DT. Figure 5A shows B16 tumors are repopulated by cDCs after termination of DT treatment. Analysis of the frequency of repopulating cells demonstrated that tumors previously depleted of CD11c^+^ cells contained an increased frequency of cDC1 and cDC2 because of an increase in cells within the CD24^low to high^CD64^neg^ total cDC gate (Figure 5B). Total numbers of cDCs were also increased following repopulation of DT-treated tumors, but we found large variability in cDC numbers between experiments, and these differences did not reach statistical significance (Figure 5C). This response was specific to the cDC niche because we did not observe an increase in monocyte or macrophage populations in repopulating tumors (Figure 5C).

To test whether newly recruited cDC1 had the potential to activate recruited TRP-2 TCR-transduced T cells, we investigated whether repopulating cDC1 cross-presented endogenous tumor antigen to TRP-2 T cells. Thus, we purified CD11c^+^ cells from tumors growing in CD11c.DTR chimeras 1 week after the last injection of PBS or DT. Isolated CD11c^+^ cells were cultured with freshly transduced TRP-2 T cells, which were assessed 18 hr later for antigen-specific interferon γ (IFN-γ) production. Figure 5D shows that there was no difference in the ability of TRP-2 T cells to produce IFN-γ in response to peptide-pulsed cells from either PBS- or DT-treated mice, suggesting no overt intrinsic differences between resident and repopulating cDCs. However, in the absence of exogenous antigen, purified cDCs from DT-treated mice were more effective in stimulating IFN-γ production of TRP-2-specific T cells, suggesting improved presentation of naturally processed peptide.

Therefore, together, these data suggest that depletion of CD11c^+^ cells drives the recruitment and/or expansion of cDCs in B16 tumors, and that repopulating cDC1 process and cross-presents tumor antigen to TRP-2 T cells.

### Injection of DT Depletes a Population of EGFP^+^ Tumor Macrophages from B16 Tumors

**Figure 6:**
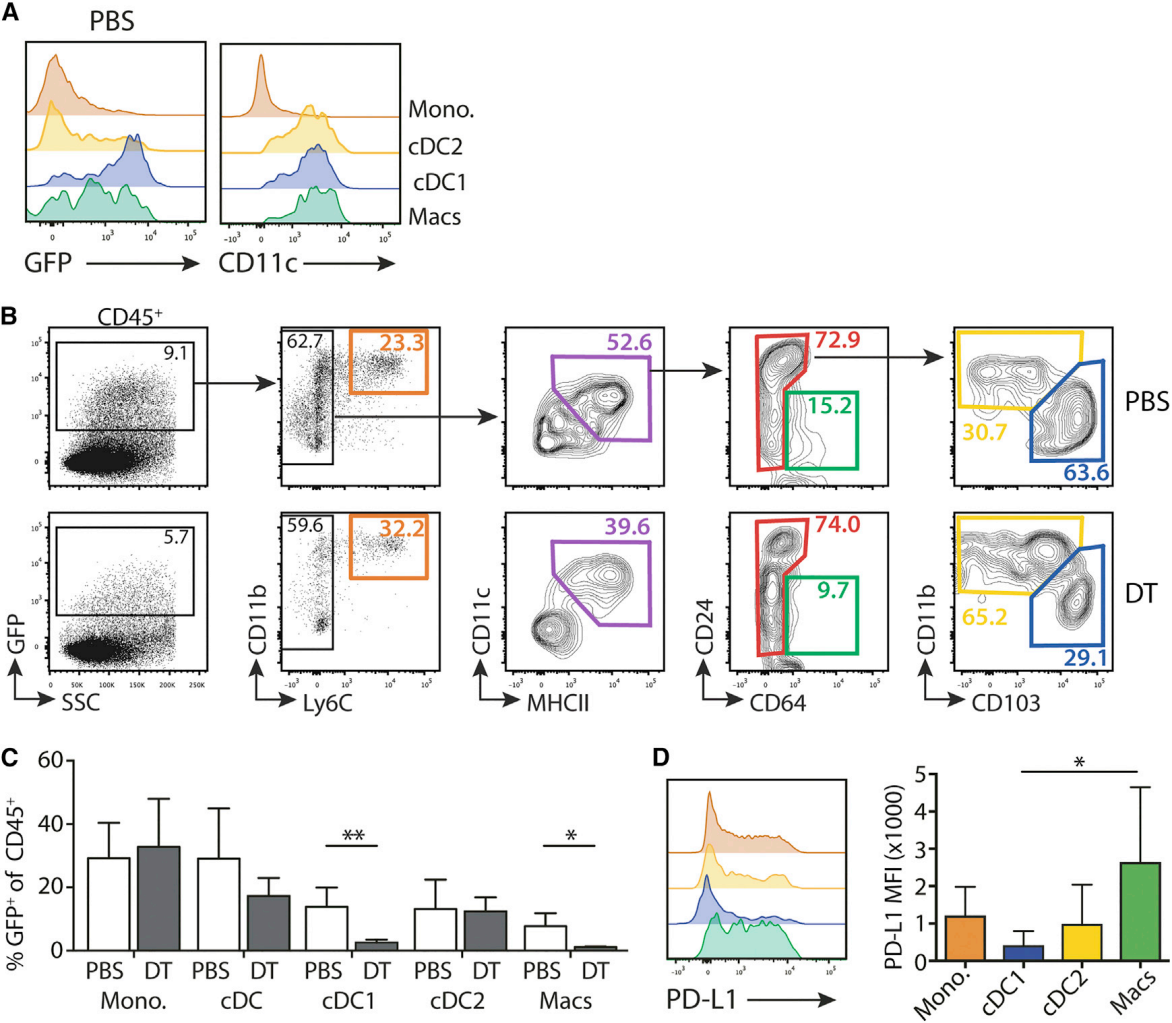
CD11c.DTR-Linked Reporter EGFP Expression Identifies a Subset of Tumor-Resident Macrophages that Are Sensitive to DT (A) Representative histograms showing expression of EGFP or CD11c expression in tumor myeloid populations of control CD11c.DTR mice. Histogram counts are normalized to mode. (B) Representative contour plots showing gating of live, single CD45^+^ cells 48 hr after a single injection of PBS or DT. (C) Bar graph showing the frequency (±SD) of gated cells within the EGFP^+^ population either in PBS controls or the non-depleted EGFP^+^ cells in DT-treated mice. Mono., monocyte; Macs, macrophages (CD64^+^). PBS versus DT cDC1 p = 0.0099; macrophages p = 0.0176; n = 4 from one experiment. (D) Left: representative histograms of PD-L1 expression on myeloid cells within B16 tumors. Right: bar chart showing median PD-L1 fluorescence (±SD). cDC1 versus macrophages p = 0.05; n = 5 from two independent experiments. ^∗^p < 0.05, ^∗∗^p < 0.01.

The aberrant activity of the CD11c.DTR transgene can lead to DT-mediated deletion of unexpected cell types outside the DC lineage. The transgenic construct that was used to generate the CD11c.DTR mice contains EGFP as a reporter gene.^26^ This provides an opportunity to identify cells in which the CD11c.DTR construct is active, which may render them susceptible to DT-mediated deletion. We, therefore, analyzed in detail the EGFP expression profile of hematopoietic cells present in B16 tumors. Comparison of endogenous CD11c expression and CD11c.DTR EGFP transgene expression demonstrated clear differences in the expression profiles of tumor myeloid cells (Figure 6A). Thus, while cDC1 and cDC2 expressed similarly high levels of endogenous CD11c, the EGFP expression was remarkably different, with high levels in cDC1 and low levels in cDC2. This correlated with the previously described differential sensitivity of the two cDC subsets to depletion with DT (Figure 1). Although all macrophages expressed high levels of endogenous CD11c, a substantial proportion was negative for EGFP expression. This observation explained the resistance of some tumor macrophages to DT in these mice (Figure 1). In Figure 6B, 8.5% ± 0.64% (SEM) of live CD45^+^ tumor-infiltrating cells expressed EGFP, and injection of DT reduced this to 3.3% ± 0.43% (n = 4). Flow cytometric analysis of cells remaining within the CD45^+^EGFP^+^ gate after treatment with DT demonstrated that, while EGFP^+^ cDC1 and macrophages were efficiently ablated, EGFP^+^ cDC2 and monocytes appeared unaffected and remained in the tumor (Figures 6B and 6C). Therefore, this analysis revealed differences in the activity of the DTR transgene within different CD11c^+^ populations and demonstrated the preferential loss of a subset of EGFP^+^ DT-sensitive macrophages. Further analysis did not reveal any phenotypic differences between macrophages expressing high or low levels of EGFP. However, staining for the immunosuppressive ligand programmed death-ligand (PD-L)-1 demonstrated that tumor macrophages expressed high levels of this molecule (Figure 6D). Therefore, these data suggest that depletion of local tumor macrophages, in the context of the augmented cDC1 niche, may contribute to the expansion of transferred T cells in DT-treated mice.

## Discussion

TCR gene therapy is a potent therapeutic strategy for cancer, leading to durable cancer regression in some patients in the absence of some of the toxicities caused by other T cell immunotherapies. Transfer of *in vitro*-activated TCR-transduced T cells bypasses a requirement for priming by the patient’s endogenous cDCs. However, it was not clear whether interactions between transferred effector T cells and cDCs within the tumor were required or, indeed, sufficient for the expansion and survival of therapeutic T cells. Here, we show that tumor cDC1 can engage TCR-engineered T cells and contribute to their expansion in the tumor microenvironment. But this occurs in the context of local macrophages, which have the potential to cause suppression of T cell function. Together, these data demonstrate the importance of endogenous myeloid cells within the tumor microenvironment in regulating recruited TCR-gene-modified T cells.

cDC1 are specialized cross-presenting cells and are highly immunogenic within the tumor environment.^5^, ^8^ We have previously exploited the temporal depletion of CD11c^+^ cells from CD11c.DTR mice to demonstrate the potency of repopulating cDCs in LNs to cross-present lentiviral antigen in a vaccination immunotherapy model.^22^ Here, we demonstrated that repopulation of cDCs within the tumor microenvironment resulted in the relative augmentation of both tumor cDC1 and cDC2 populations. This was associated with enhanced presentation of endogenous tumor antigens to TRP-2 TCR T cells and increased accumulation of more differentiated CD62L^neg^ TCR-engineered T cells. However, we also observed depletion of some tumor-resident macrophages that potentially expressed higher levels of the inhibitory ligand, programmed death ligand (PD-L) 1, than other myeloid populations. Therefore, together, our data suggest a working model in which cDC1 engage infiltrating engineered T cells to promote differentiation and expansion in the tumor microenvironment, but these interactions occur in the context of inhibitory signals from local macrophages and, possibly, other myeloid cells. In support of this model, Wrzesinski et al.^27^ demonstrated that transplantation of hematopoietic stem cells into irradiated mice resulted in expansion of transferred *in vitro*-activated CD8 T cells, but this was not sufficient to mediate tumor rejection in the absence of myeloablative conditioning to remove endogenous regulatory cells. We also found that, while recruited cDCs enhanced T cell numbers and activation within tumors, it was not sufficient to enhance tumor rejection in the poorly immunogenic B16 model (C.L.B. and H.J.S., data not shown).

Pre-DCs are recruited to tumors,^28^ and it has recently been shown that the growth factors that control cDC development in the tissues are also required for development of the tumor-resident DC compartment.^8^ However, melanomas evolve intrinsic mechanisms, such as increased signaling via β-catenin or secretion of prostaglandin E_2_ (PGE_2_), to selectively block recruitment of cDC1.^9^, ^18^ Our work suggests that, in addition to these tumor-intrinsic mechanisms, the tumor hematopoietic compartment also determines the composition of resident myeloid cells, and this can be re-set upon depletion of CD11c^+^ cells. The mononuclear phagocytic system is a highly dynamic cellular compartment with the plasticity to rapidly respond to changing levels of circulating growth factors. Depletion of CD11c^+^ cells has been shown to result in a rapid increase in myeloid serum growth factors, including G-CSF and fms-like tyrosine kinase (Flt) 3 ligand.^29^ Under these conditions, repopulation by DCs is preceded by a transient monocytosis in the BM, blood, and spleen, but not the skin.^24^ Our data indicate that systemic changes to myeloid growth factors do not lead to accumulation of monocytes or neutrophils within B16 tumors, but they suggest the possibility that local alteration of the tumor microenvironment, for example, due to a loss of immunosuppressive macrophages, regulates the population of the tumor cDC niche. Thus, we propose that, during normal growth, regulation of engineered T cells by tumor macrophages may dominate. However, interventions to alter the population dynamics, such that new cDC1 are recruited into the tumor, may shift this balance to enhance T cell accumulation.

Vaccination is essential for the therapeutic efficacy of gene-modified T cells in a prostate cancer model,^30^ and this, with observations that TCR-transduced T cells are detected in the draining, but not the non-draining LNs of tumor-bearing recipients,^31^ has led to an assumption that gene-modified T cells require stimulation by cDCs *in vivo* to enhance their therapeutic activity.^32^ However, these data focus on the role of cDCs in lymphoid organs, and the site of cDC-T cell interaction in tumor-bearing recipients has not been carefully investigated. Our data demonstrate that *in vitro-*activated T cells preferentially accumulate in tumors, but not LNs or spleen, inferring a role for local interactions with tumor-resident myeloid cells. Recent data have suggested that anti-tumor activity by adoptively transferred cytokine-activated TCR transgenic T cells depends on recruitment of cDC1 to tumors.^9^ We show that baseline recruitment of engineered T cells that have been activated *in vitro* via their TCRs was not impaired in the absence of cDCs. Together, these studies highlight the importance of cDC1 for T cell immunotherapy, but they reveal potential differences in the nature of the interactions between endogenous cDCs and the different types of T cells used for ACT.

Gene-engineered therapies using T cells that express tumor-specific TCRs or CARs have shown success in blood cancers, but their potential in solid tumors has hampered the immunosuppressive microenvironment. We and others have demonstrated that genetic modification of TCR constructs to enhance functional avidity may augment T cell fitness within the tumor,^33^, ^34^, ^35^, ^36^ but the therapeutic potential of these cells is limited by factors that control recruitment, survival, and activation of tumor-infiltrating T cells. Our findings on the importance of tumor cDCs have clinical implications for their targeted activation to enhance engineered T cell function within the tumor environment. One possibility is that this would also augment tumor antigen spread by DCs to broaden the activation of endogenous anti-tumor T cells, as has recently been shown after administration of a combination therapy for large established B16 melanomas.^37^ The data presented here highlight the importance of the balance between beneficial and inhibitory interactions with endogenous myeloid cells to control expansion of engineered T cells within the tumor microenvironment and point toward future strategies that manipulate endogenous myeloid cells to promote therapeutic responses to solid organ tumors.

## Materials and Methods

### Mice and Tumor Injection

Animals were used under protocols approved by local institutional research committees and in accordance with UK Home Office guidelines. C57BL/6 (B6) mice were bred in-house. Homozygous CD11c.DTR (B6.FVB-Tg(Itgax-DTR/ EGFP)57Lan/J) mice were bred in-house. Rag2^−/−^ mice were bought from Jackson Laboratory and crossed in-house to CD11c.DTR mice. CD11c.DTR and CD11c.DTR.Rag2^−/−^ syngeneic chimeras were generated as described previously.^38^ CD11c^+^ cells were depleted upon intraperitoneal injection of 100 ng of DT (Sigma, UK) in PBS according to published protocols. Chimeras received single injections or repeated injections once every 72 hr, and depletion was assessed in control animals 24–48 hr after injection of DT.

B16.F10 melanoma cells were cultured in RPMI 1,640 medium (Lonza), supplemented with 10% fetal calf serum (FCS; Biosera), 1% L-glutamine (2 mM), and 1% penicillin/streptomycin (100 U/mL). C57BL/6 mice were injected subcutaneously with 1 × 10^5^ B16.F10 cells in 100 μL of PBS. Tumors became palpable by days 5–10. Tumor size was assessed using calipers every 2–3 days. Mice were euthanized if tumors grew to >15 mm in any direction, as per home office regulations.

### Generation of TRP-2 TCR-Transduced T Cells

The TRP-2 TCR, which recognizes TRP-2_181–188_, was a kind gift from Professor Ton Schumacher (Netherlands Cancer Institute) and was cloned into the retroviral vector pMP71 with a 2A sequence separating the Vα12.1 and Vβ3 chains, followed by an internal ribosome entry site (IRES) truncated CD19 sequence.^39^ The TCR was codon optimized and also contains an extra cysteine residue in the constant chains to enhance pairing of the α and β chains.^40^, ^41^

To generate TRP-2 retroviral particles, Phoenix-Eco (PhEco)-adherent packaging cells (Nolan Laboratory) were transiently transfected with retroviral vectors for the generation of supernatant containing the recombinant retrovirus required for infection of target cells, as described previously.^33^ The PhEco-adherent packaging cells were transfected using Fugene HD (Roche) with the pCL-eco construct and the TRP-2 TCR vector according to the manufacturers’ instructions. CD8^+^ T cells were purified from C57BL/6 splenocytes by magnetic selection according to the manufacturer’s instructions (Miltenyi Biotec). Sorted cells were activated with concanavalin (Con) A (2 μg/mL) and IL-7 (1 ng/mL) for 24 hr, and then 4–6 × 10^6^ activated T cells were incubated for a further 24 hr with retroviral particles on retronectin-coated (Takara-Bio) 6-well plates, in the presence of IL-2 (100 U/mL; Roche). Transduced cells were injected intravenously into mice 24 hr after transduction.

### Lymphocyte Isolation from Tumors

Tumor samples were mechanically digested using dissection scissors before culturing in 1 mL of RPMI containing 320 μg of Liberase TL (Roche) and 200 μg of DNase 1 grade 2 (Roche). These were incubated at 37°C in a shaking incubator for 30 min. Tumor leukocytes were then isolated as interphase cells after centrifugation with Histopaque-1077 (Sigma-Aldrich).

### Flow Cytometry

The following monoclonal antibodies were used to stain cells: anti-CD11b v450, F4/80 APCeFluor780, Ki67 eFluor660, CD19 PerCpCy5.5, Thy1.1 PeCy7 (eBiosciences), CD11c fluorescein isothiocyanate (FITC) or allophycocyanin (APC), CD24 BV711, CD45.2 v500, CD103 biotin, I-Ab phycoerythrin (PE), Ly6C PeCy7, PD-L1 APC, streptavidin PerCpCy5.5, CD4 APC-H7, CD8 v450, CD62L Alexa700, IFN-γ APC, Vβ3 PE (BD Biosciences), or CD64 PE (BioLegend). Exclusion of propidium iodide was used to gate live cells. Cells were enumerated using CountBright absolute counting beads (Thermo Fisher Scientific). Samples were acquired using the Fortessa flow cytometer (BD Biosciences) and analyzed using FlowJo software (Treestar).

### Multidimensional Reduction Analysis

The automated t-SNE algorithm was used to visually analyze fluorescence-activated cell sorting (FACS)-stained lymphocytes (viSNE).^19^ This was performed using the Cytobank online platform.^42^ Cells were pre-gated on live (PI^−^) CD45^+^ cells before analysis. Defined populations were then characterized by conventional FACS analysis and overlaid over the viSNE coordinates where indicated.

### T Cell Restimulation Assay

RMA/S cells are a transporter associated with antigen processing (TAP)-deficient derivative of the RMA cell line that can efficiently present exogenously loaded peptide. TRP-2-TCR-transduced T cells were rested for 5 days post-transduction, then stimulated with peptide loaded cells. To stabilize empty major histocompatibility (MHC) molecules, 1 × 10^6^ RMA/S cells were cultured overnight at 20°C. The next day, RMA/S cells were incubated with 10 μM–100 pM of relevant (TRP-2_181–188_: VYDFFVWL) or irrelevant (gp100_25–33_: EGSRNQDWL) peptide at 37°C for 3 hr. RMA/S cells were then irradiated with 80 Gy and washed in complete RPMI, and 1 × 10^5^ cells were plated in a 96-well plate for 18 hr with 1 × 10^5^ TRP-2 TCR-transduced T cells. Intracellular IFN-γ was detected in transduced T cells using the BD Perm/fix kit with brefeldin A according to the manufacturer’s instructions (BD Biosciences) and stained with anti–IFN-γ antibody (BD Biosciences). Alternatively, 1 × 10^5^ irradiated (80 Gy) B16.F10 or EL4 tumor cells were cocultured overnight with 1 × 10^5^ TRP-2 TCR-transduced T cells, and IFN-γ expression was assessed as indicated above.

### Restimulation with Tumor CD11c^+^ Cells

CD11c^+^ cells were positively selected from tumor leukocytes by incubation with murine anti-CD11c MicroBeads and two MS magnetic separation columns (Miltenyi). CD11c^+^ cells were peptide loaded, or not, with 1 μM relevant peptide (TRP-2_181–188_: VYDFFVWL) for 2 hr, then washed and plated in a 96-well plate. 1 × 10^4^ CD11c^+^ cells were cocultured overnight with 1 × 10^5^ TRP-2TCR-transduced T cells. After 18 hr, cells were incubated with brefeldin A (eBioscience) and stained for intracellular IFN-γ as indicated above.

### Statistical Analysis

Statistical comparisons were made by using a two-tailed Student’s t test for parametric data, a two-tailed Mann-Whitney U test for non-parametric data, and a Wilcoxon signed rank test for comparing relative values to the mean of the control.

## Author Contributions

Conceptualization, C.L.B. and H.J.S.; Methodology, A. Hotblack, C.L.B., and H.J.S.; Investigation, A. Hotblack, A. Holler, A.P., and S.W.; Writing – original draft, A. Hotblack and C.L.B.; Writing – review and editing, A. Hotblack, H.J.S., and C.L.B.; Funding Acquisition, H.J.S. and C.L.B.

## Conflicts of Interest

H.J.S. is the scientific advisor and shareholder of Cell Medica and receives research funds from Cell Medica.
